# Adult care for Duchenne muscular dystrophy in the UK

**DOI:** 10.1007/s00415-014-7585-3

**Published:** 2014-12-24

**Authors:** Sunil Rodger, Katherine L. Woods, Catherine L. Bladen, Angela Stringer, Julia Vry, Kathrin Gramsch, Janbernd Kirschner, Rachel Thompson, Katharine Bushby, Hanns Lochmüller

**Affiliations:** 1 Institute of Genetic Medicine, International Centre for Life, Newcastle University, Central Parkway, Newcastle upon Tyne, NE1 3BZ UK; 2 Action Duchenne, The Epicentre, 41 West Street, London, E11 4LJ UK; 3 Clinic II: Division of Paediatric Neurology and Muscular Diseases, Department of Paediatrics and Adolescent Medicine, University Medical Center Freiburg, Mathildenstraße 1, 79116 Freiburg, Germany

**Keywords:** Duchenne muscular dystrophy, DMD, Care, Adult, Neuromuscular, CARE-NMD

## Abstract

Survival in Duchenne muscular dystrophy (DMD) has increased in recent years due to iterative improvements in care. We describe the results of the CARE-NMD survey of care practices for adults with DMD in the UK in light of international consensus care guidelines. We also compare the UK experience of adult care with the care available to pediatric patients and adults in other European countries (Germany, Denmark, Bulgaria, Czech Republic, Hungary, and Poland). UK adults experience less comprehensive care compared to children in their access to specialized clinics, frequency of cardiac and respiratory assessments, and access to professional physiotherapy. Access to the latter is especially poor when compared to other European adult cohorts. Although the total number of nights in hospital (planned and unplanned admissions) is lower among UK adults than elsewhere in Western Europe, social inclusion lags behind other Western European countries. We observe that attendance at specialized clinic is associated with more frequent cardiac and respiratory assessments among adults, in line with international best practice. Attendance at such clinics in the UK, though comparable to other countries, is still far from universal. With an increasing adult population living with DMD, and cardiac and respiratory failure the leading causes of death in this population, we suggest the need for an urgent improvement in adult access to specialized clinics and to consistent, comprehensive best practice care.

## Introduction

Duchenne muscular dystrophy (DMD) is a severe neuromuscular disease affecting one in 3,600–6,000 live male births [1, 2], characterized by progressive muscle weakness which causes loss of ambulation by the early teenage years. Cardiac and respiratory complications are seen, especially with disease progression, and if untreated DMD typically leads to death in the late teens [3]. Although currently incurable, the natural history of DMD has been improved by targeted interventions addressing known disease complications. Internationally generated and accepted consensus care guidelines for DMD have been published and have achieved NICE process accreditation in the UK [3–5]. Multidisciplinary care, particularly the initiation of corticosteroids and assisted ventilation supported by regular cardiac and respiratory monitoring, has led to increasing numbers of patients living into adulthood in recent years (Bladen, manuscript in preparation) [6–8]. This adult population requires continued specialist multidisciplinary care. Cardiac and respiratory complications are the primary cause of death in DMD, and regular monitoring of heart and lung function is an important pillar of best practice care for adults [4]. It is known that transition from childhood to adulthood poses a range of challenges in DMD and other chronic conditions with complex healthcare needs, relating both to the transfer of medical care and to the wider process of societal transition and integration [9–12]. Reports suggest that care for DMD across all age groups varies significantly both within and between countries, including in the UK [13]. However, quantitative data has so far been lacking, particularly for the adult population.

Within the 3-year EU-funded CARE-NMD project [14], we have been able to study the experience of care in DMD in seven European countries (Kirschner, manuscript in preparation). Given the progressive nature of the condition and the increasing numbers of people with DMD living into adulthood, it is timely to consider the extent to which the care received by adults with DMD is in line with international consensus recommendations. Using data collected during the CARE-NMD patient survey of care practices, this study investigates the care received by UK adults and compares it to the recommended care guidelines, the care received by pediatric patients in the UK, and the care received by the adult cohorts in other countries surveyed.

## Methods

The CARE-NMD project conducted a cross-sectional, multi-center study surveying care practices and quality of life in DMD in seven European countries: Bulgaria, the Czech Republic, Denmark, Germany, Hungary, Poland, and the United Kingdom (Kirschner, manuscript in preparation). The project collected self-report data via surveys of patients/families with DMD and neuromuscular clinics treating those with the condition.

This paper describes adult results from the two-part patient and family questionnaire, which examined the care received and quality of life experienced by those living with DMD. The 42-question care survey captured socio-demographic variables, as well as information about daily life, functional abilities, disease progression, and medical and social care received. The survey was designed by project partners to determine the extent to which participants received the care outlined in the consensus care recommendations. It was translated into the local language by project partners in each country, and piloted on between five and ten patients and their families in each country for comprehensibility. Any adjustments were then reconciled with the original English version before full distribution. The quality of life section used existing validated health-related quality of life (HRQoL) instruments. PedsQL Generic and Neuromuscular modules, WHO-QOL BREF, and SF-36 were chosen based on criteria including existing validation in most project languages, availability of reference values for most participating countries, previous use in studying DMD, and inclusivity of ages from 2 years to adulthood. The range of instruments permitted the utilization of age-appropriate HRQoL surveys for all participant age groups: for those under 18 years, both patient- and parent-report surveys were distributed^1^.

Combined questionnaire packs were distributed in each country on behalf of the project via national DMD patient registries, which form part of the TREAT-NMD Global DMD Registries [15]. This ensured that age-appropriate HRQoL surveys were sent while preserving respondent anonymity, ensuring the CARE-NMD project team could not identify any individual. Links to electronic questionnaires, hosted on SurveyMonkey, were distributed electronically where e-mail addresses were available; paper versions were sent in other cases, or where no response or opt-out was received electronically. Data collection took place between September 2011 and April 2012, with paper versions input into the SurveyMonkey questionnaire by the CARE-NMD project leaders at University Medical Center Freiburg. Data analysis was carried out in IBM SPSS and Microsoft Excel.

## Results

We present here the results on the care received by adults with DMD in the UK in the wider context of the experience of under-18s in the UK, and of adults in the other countries surveyed. In particular, in these comparisons we have mainly focused on Denmark and Germany due to comparable cohorts, and similar characteristics as Western European states with robust healthcare systems. Future manuscripts will examine other data from the CARE-NMD survey, including Eastern European countries, quality of life, and child cohorts.

### Demographics

1,062 valid responses were received from 1,677 surveys distributed (63.3 % response rate). Both the proportion and absolute number of adult responses varied considerably between countries, and were lower in Eastern than Western Europe (Table 1). 226 responses (21.3 %) were from UK patients, with 42 (18.6 %) aged over 18. Although the overall UK response rate was the lowest in Western Europe, the proportion of adults who responded in the UK (18.6 %) was similar to Germany (18.3 %). UK responses made up 20.9 % of the adult responses, comparable to Denmark (21.4 %).

**Table 1:** Adult respondents to CARE-NMD patient and family survey

Country	Sent	Responses (% rate)	Responses 18+	Adult responses per country (%)	Overall adult cohort (%)
UK	421	226 (53.7)	42	18.6	20.9
Germany	545	420 (77.1)	77	18.3	38.3
Denmark	131	88 (67.2)	43	48.9	21.4
**Western Europe**	**1,097**	**734 (66.9)**	**162**	**22.1**	**80.6**
Bulgaria	73	40 (54.8)	7	17.5	3.48
Hungary	70	57 (81.4)	5	8.8	2.49
Poland	246	142 (57.7)	16	11.3	7.96
Czech Republic	191	89 (46.6)	11	12.4	5.47
**Eastern Europe**	**580**	**328 (56.6)**	**39**	**11.9**	**19.4**
**Overall**	**1,677**	**1,062 (63.3)**	**201**	**18.9**	**100**

The age distribution of the UK adult cohort was weighted toward younger adults, with 35 (83.3 %) aged under 27 (Table 2). This was similar to Germany (81.8 %), though considerably different from Denmark where 44.2 % were aged under 27, and more than one-third over 33 years old. The age distribution of adults in Eastern Europe was also significantly weighted toward younger adults, with the majority in all countries except Poland aged 18–22.

**Table 2:** Age distribution of adult respondents to CARE-NMD Survey by country

Country	18–22	23–27	28–32	33+
UK	21	14	4	3
Germany	46	17	9	5
Denmark	10	9	9	15
**Western Europe**	**77**	**40**	**22**	**23**
Bulgaria	5	1	1	0
Hungary	4	1	0	0
Poland	7	5	3	1
Czech Republic	10	1	0	0
**Eastern Europe**	**26**	**8**	**4**	**1**
**Overall**	**103**	**48**	**26**	**24**

With a mean age of 24.1 years, the UK adult cohort was slightly younger than that across all countries (24.8 years). All were non-ambulatory: mean UK loss of ambulation was 10.4 years, somewhat younger than the overall adult cohort of 11.1 years. Average age at diagnosis was 4.6 years, the lowest of the adult cohorts, and a little more than 6 months younger than the mean.

**Fig. 1:**
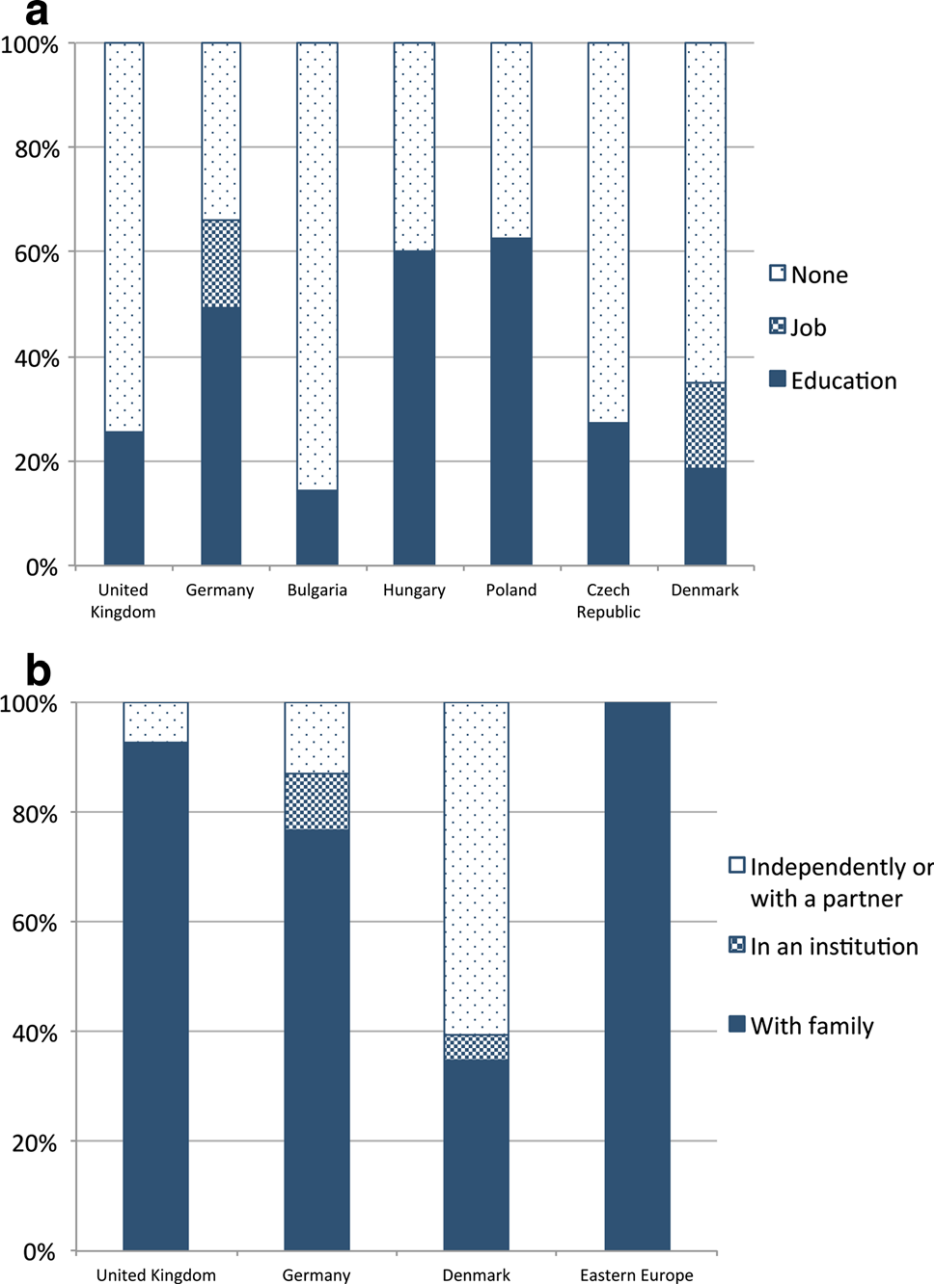
Demographics of adult cohort: **a** occupational status of adults with DMD by country, **b** living arrangements of adults with DMD by country

None report having a job, with 25.6 % still in education (secondary school, special needs school, vocational training, or university). Occupational status contrasted starkly with Germany, where 49.4 % were in education and a further 16.9 % were employed. The proportion of UK adults in education was comparable to that of Denmark (18.6 % in education), though a further 16.3 % were in employment in that country. In the four Eastern European countries^2^, although none of the 39 adults surveyed were in employment, 17 (43.6 %) were in education (Fig 1a).

A similar pattern emerged with living arrangements, where the proportion of UK adults living independently (7.1 %) was lower and the proportion living at home (92.9 %) higher than elsewhere in Western Europe. No adults in the UK reported living with a partner. In Denmark, 60.5 % lived independently or with a partner and 34.9 % lived with their family, while in Germany these figures were 13.0 and 76.6 %, respectively. In Eastern European countries, all adults reported living at home (Fig 1b). More than three times as many adults reported not getting out of the house at all in a typical week in the UK (28.6 %) than in Denmark (9.3 %).

### Satisfaction and specialized clinic experience

UK adult satisfaction with care was lower than elsewhere in Western Europe, while dissatisfaction is higher; three-quarters of UK adults said they were “very” or “rather” satisfied (Table 3). Adult satisfaction in the UK was also lower and dissatisfaction higher than in the UK pediatric cohort (86.5 and 13.5 %, respectively). Satisfaction among German adults was comparable to the UK pediatric cohort, while Danish adults were overwhelmingly satisfied with their care.

**Table 3:** Overall satisfaction with care (UK adults, UK children, German adults, and Danish adults)

Satisfaction (%)	UK (18+)	UK (<18)	Germany (18+)	Denmark (18+)
Very satisfied	8 (19.5 %)	49 (28.8 %)	16 (22.5 %)	21 (51.2 %)
Rather satisfied	23 (56.1 %)	98 (57.6 %)	46 (64.8 %)	18 (43.9 %)
**Satisfied**	**31 (75.6 %)**	**147 (86.5 %)**	**62 (87.3 %)**	**39 (95.1 %)**
Rather dissatisfied	9 (22.0 %)	20 (10.9 %)	8 (11.3 %)	2 (4.9 %)
Not satisfied at all	1 (2.4 %)	3 (1.6 %)	1 (1.4 %)	0 (0 %)
**Dissatisfied**	**10 (24.4 %)**	**23 (13.5 %)**	**9 (12.7 %)**	**2 (4.9 %)**

International consensus DMD care guidelines recommend multidisciplinary care managed by a disease expert, with co-ordinated interventions from other specialities as required. Such care, particularly during childhood, is often delivered via specialized neuromuscular clinics. Indeed, the loss of the pediatric family centric approach during transition to adult clinics has been identified as a challenge for young people with DMD [12]. UK families highly value the continuity provided by a transition model continuing their access to the same specialized team [16].

**Fig. 2:**
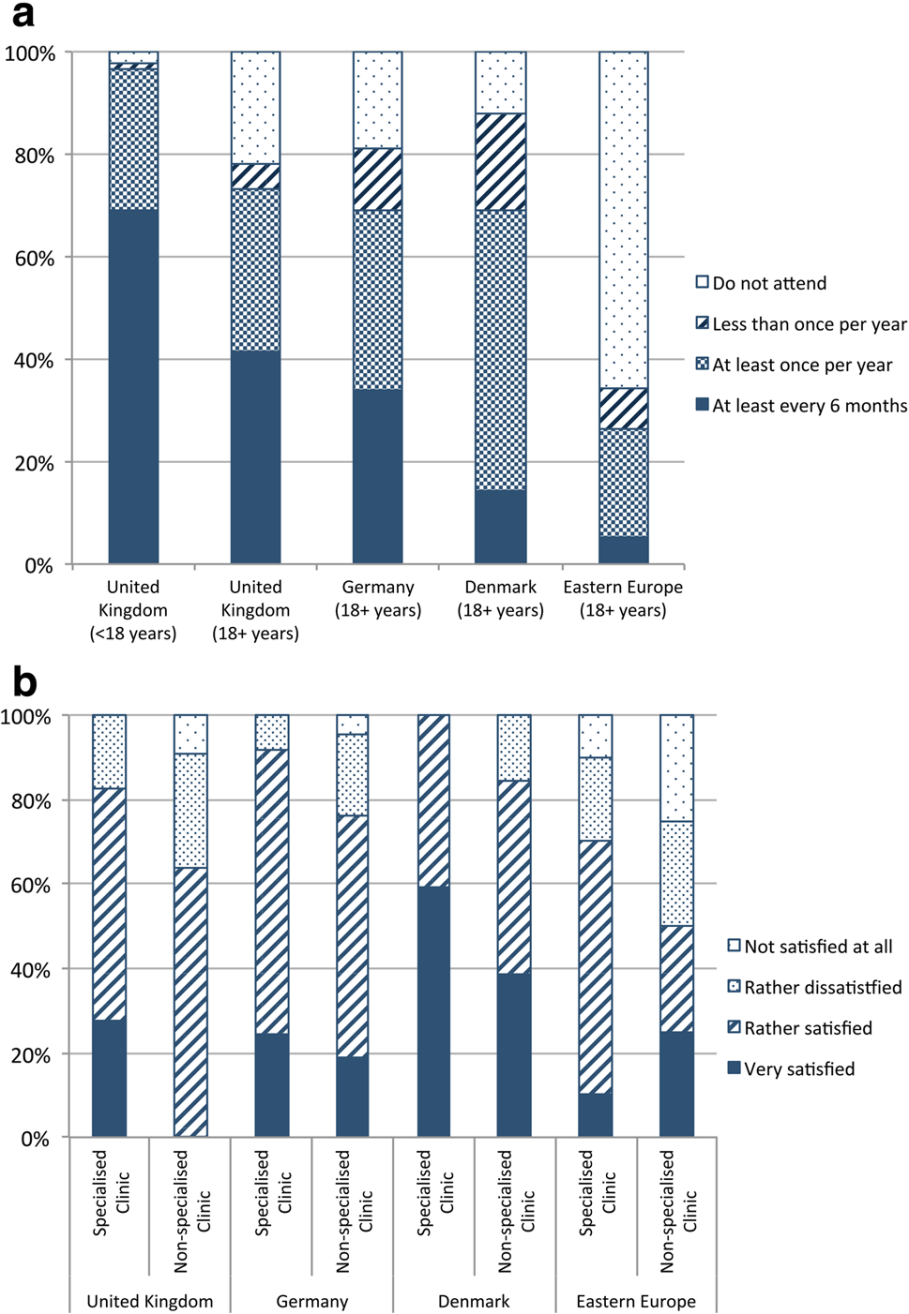
Neuromuscular clinic experience **a** attendance at specialized clinic. **b** Satisfaction with care among adults who attend a specialized clinic annually and those who do not

73.2 % of UK adults attend what they recognized as a specialized clinic at least once annually, with 41.5 % of these attending at least once every 6 months. Both figures were considerably lower than the UK pediatric cohort (96.6 % with 69.0 % attending every 6 months). At least annual attendance was slightly higher than adult cohorts in Germany (68.9 %) and Denmark (69.0 %), and significantly higher than Eastern Europe (25.6 %). However, of the three Western European countries, UK adults reported the highest rate of complete non-attendance at a specialized clinic (22 %), compared to 11.9 % in Denmark and 18.9 % in Germany. Complete non-attendance in Eastern Europe ranged between 85.7 % (Bulgaria) and 40 % (Hungary), with a mean of 64.1 % (Fig 2a).

Adults who attended a specialized clinic at least annually were more satisfied with their care than those who did not (Fig 2b). Of eight UK adults who claimed to be “very satisfied”, all attended a specialized clinic annually and seven (87.5 %) attended at least every 6 months. Among those who were dissatisfied, two attended a specialized clinic every 6 months while four attended less than annually; three of the latter never attended, including the only individual who was “not satisfied at all”. A link between specialized attendance and satisfaction was also found in the other countries and in the UK pediatric cohort. All seven UK adults who never attended a specialized clinic and who also provided a reason reported “distance” for their non-attendance. This reason was not reported by any of the four pediatric patients who did not attend a center.

Staff at a specialist center should not only be familiar with best practice care, but also provide appropriate information to DMD patients about their condition and treatment options. We asked whether respondents felt the information they received was sufficient across several important areas of care for adults: the course of the disease and main problems that may arise; the use of steroids; breathing problems; and cardiac problems. Responses were compared across three groups: adults not seen at specialized clinics annually, and both adults and children seen annually.

UK adults who did not attend a specialized clinic annually reported considerably lower levels of sufficient information provision across all four areas than adults who did, with a substantial difference observed about information on steroids, breathing problems, and cardiac problems. In some cases, the proportion of those receiving sufficient information among non-attendees was less than half of those seen at a specialized clinic annually (Table 4). The proportion of adults who were provided with information was lower among specialized clinic attendees than non-attendees. Non-provision of information varied by topic, but was more than 12 times (general information and complications), 9 times (breathing problems), 6 times (cardiac problems), and 3 times (steroids) lower among those seen at a specialized clinic than among those who were not.

**Table 4:** Provision of information about important aspects of DMD care to different groups in the UK

	Adults not seen at specialized clinic (%)	Adults seen at specialized clinic (%)	Children seen at specialized clinic (%)
Respondents rating the provision of information as “sufficient” in each area			
The course of the disease and the main problems that may arise	44.4	67.9	70.7
Treatment with steroids in DMD	25.0	58.6	78.9
Breathing problems in the course of DMD	44.4	85.2	46.4
Cardiac problems in DMD	33.3	69.0	56.6
Respondents who responded “no, not at all” in relation to information being provided on			
The course of the disease and the main problems that may arise	44.4	3.6	4.8
Treatment with steroids in DMD	62.5	20.7	2.4
Breathing problems in the course of DMD	33.3	3.7	22.9
Cardiac problems in DMD	22.2	3.4	13.3

Although both adults and children who were treated at a specialized clinic reported similar levels of sufficient knowledge about the disease overall, there were differences in relation to steroids, breathing problems, and cardiac problems. Sufficient information on steroid treatment was lower and those without information were higher among adults.

### Hospital admissions and care

#### Hospital admissions

172 adults provided complete information about the cause and overall duration of hospital admissions in the 2 years prior to the survey (137 from Western Europe, 34 from Eastern Europe). 40 adults in the UK provided this information with 15 (40 %) spending at least one night in hospital. On average, attendees spent 12 nights in hospital for a total of 184 overnights, of which the majority (57.6 %) were unplanned admissions. More than three-quarters (77.4 %) of these unplanned admissions were due to acute respiratory problems such as chest infection. Planned admissions in the UK were mainly for regular checkups (46.2 %) and planned surgery (48.7 %).

**Fig. 3:**
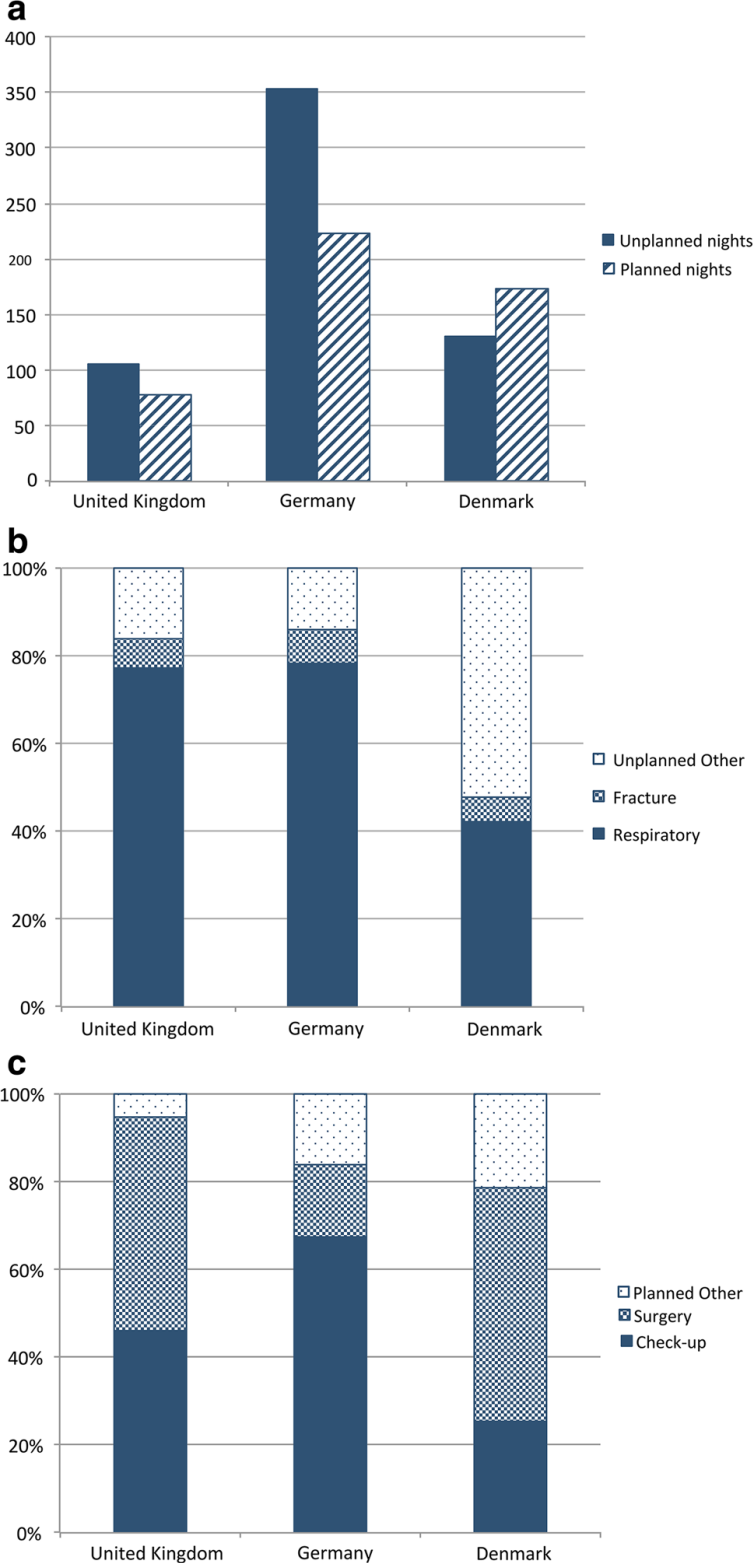
Hospital admissions of adult cohorts in Western Europe: **a** overall number of planned and unplanned nights spent in hospital by country, **b** proportion of unplanned nights spent in hospital by cause, **c** proportion of planned nights spent in hospital by cause

Fewer UK adults were admitted to hospital than in other Western European countries, and collectively they spent a lower overall number of nights as inpatients (Fig 3a). In Germany, 52.5 % of adults were admitted, recording 576 overnights (averaging 18 nights per patient admitted). 70.3 % of adults in Denmark were admitted: while the average number of nights per patient was the same as the UK (12), the overall nights recorded (304) was, therefore, considerably more.

A similar proportion of German admissions were unplanned (61.3 %) as in the UK, while in Denmark the ratio of admissions was reversed with the majority (57.2 %) being planned. In Germany, the proportion of unplanned admissions attributed to respiratory crises was similar (78.2 %) to the UK, while in Denmark it was considerably lower (42.3 %) (Fig 3b). The Danish cohort also had a much lower proportion (25.3 %) of nights in hospital attributed to regular checkups than the UK (46.2 %) and Germany (67.3 %) (Fig 3c).

Seven of ten UK adults with unplanned hospitalizations reported respiratory problems as their sole cause of admission, all of whom were mechanically ventilated. There were no clear patterns to this group: most attended a specialized clinic, received at least annual assessments of lung function, and were informed of breathing complications. There likewise appeared to be no clear association between this cause of admission and age in the UK, nor any other country surveyed.

#### Cardiovascular care

**Fig. 4:**
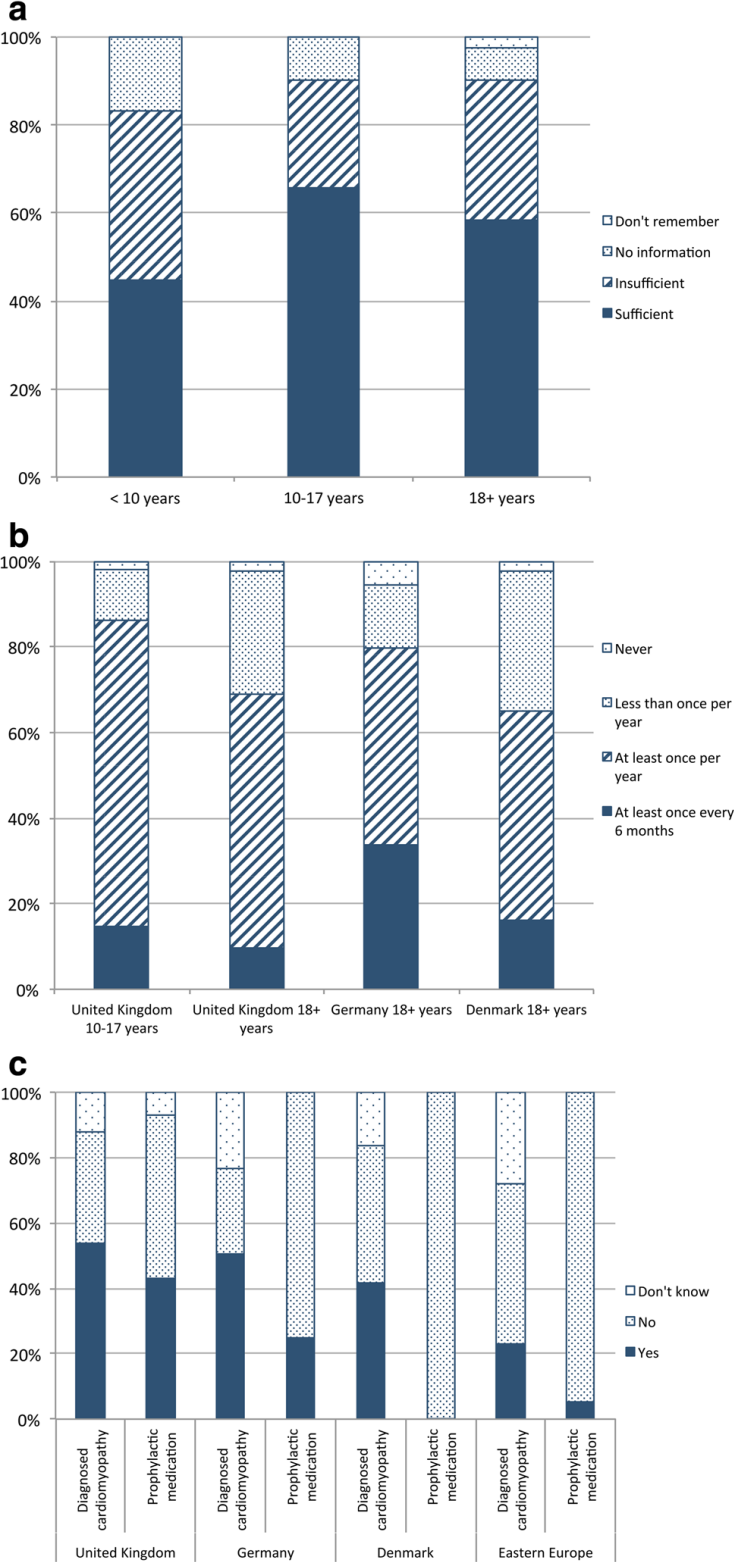
Cardiac care: **a** quality of information provided to different age groups in the UK on cardiac risks, **b** frequency of echocardiogram received by UK adults, UK children, and adults in Germany and Denmark, **c** diagnosis and prophylactic treatment of cardiomyopathy among adults by country

Information on the cardiac complications of DMD was provided to 90.2 % of UK adults, but almost one-third felt that this was insufficient and a further 7.3 % received no information. A very similar pattern was reported by Danish and German adults. “Sufficient” information was lower, and “insufficient” or “no” information higher, in Eastern Europe (Table 5). Provision of cardiac information to UK children aged 10–17 was similar to adults at 90.3 %, but a slightly higher proportion (65.6 %) rated this information “sufficient”. Of those aged under 10, 83.3 % received cardiac information with only 44.9 % rating it as sufficient (Fig 4a).

**Table 5:** Provision of information to adults about cardiac complications by country

Country (valid responses/*n*)	Provision of information about cardiac complications			
	Sufficient	Insufficient	No information	Do not remember
UK (41/42)	24 (58.5 %)	13 (31.7 %)	3 (7.3 %)	1 (2.4 %)
Germany (73/77)	51 (69.9 %)	16 (21.9 %)	3 (4.1 %)	3 (4.1 %)
Denmark (43/43)	25 (58.1 %)	13 (30.2 %)	4 (9.3 %)	1 (2.3 %)
Eastern European countries (39/42)	17 (47.2 %)	14 (38.9 %)	5 (13.9 %)	0 (0.0 %)

69.0 % of adults in the UK received at least annual echocardiograms, compared to 79.7 % in Germany and 65.1 % in Denmark. In the UK, only 9.5 % were assessed at least every 6 months, lower than in Germany (33.8 %) or Denmark (16.3 %). The proportion of adults receiving at least annual cardiac assessment was also notably lower than that of UK children aged 10–17 (86.3 %) (Fig 4b).

52.4 % of UK adults reported a diagnosis of cardiomyopathy, similar to Germany (50.5 %) and Denmark (41.9 %). There appears to be no relationship between diagnosis of cardiomyopathy and specialist clinic attendance. Almost two-thirds (65.9 %) of UK adults received medications for cardiomyopathy, of which 22.2 % did so prophylactically. Among UK adults not diagnosed with cardiomyopathy, almost half were treated prophylactically for cardiac failure, a much higher proportion than any other country surveyed: only 25 % were so treated in Germany, and none in Denmark (Fig 4c). A similar trend was evident in the UK pediatric cohort aged 10–17.

#### Respiratory care

**Fig. 5:**
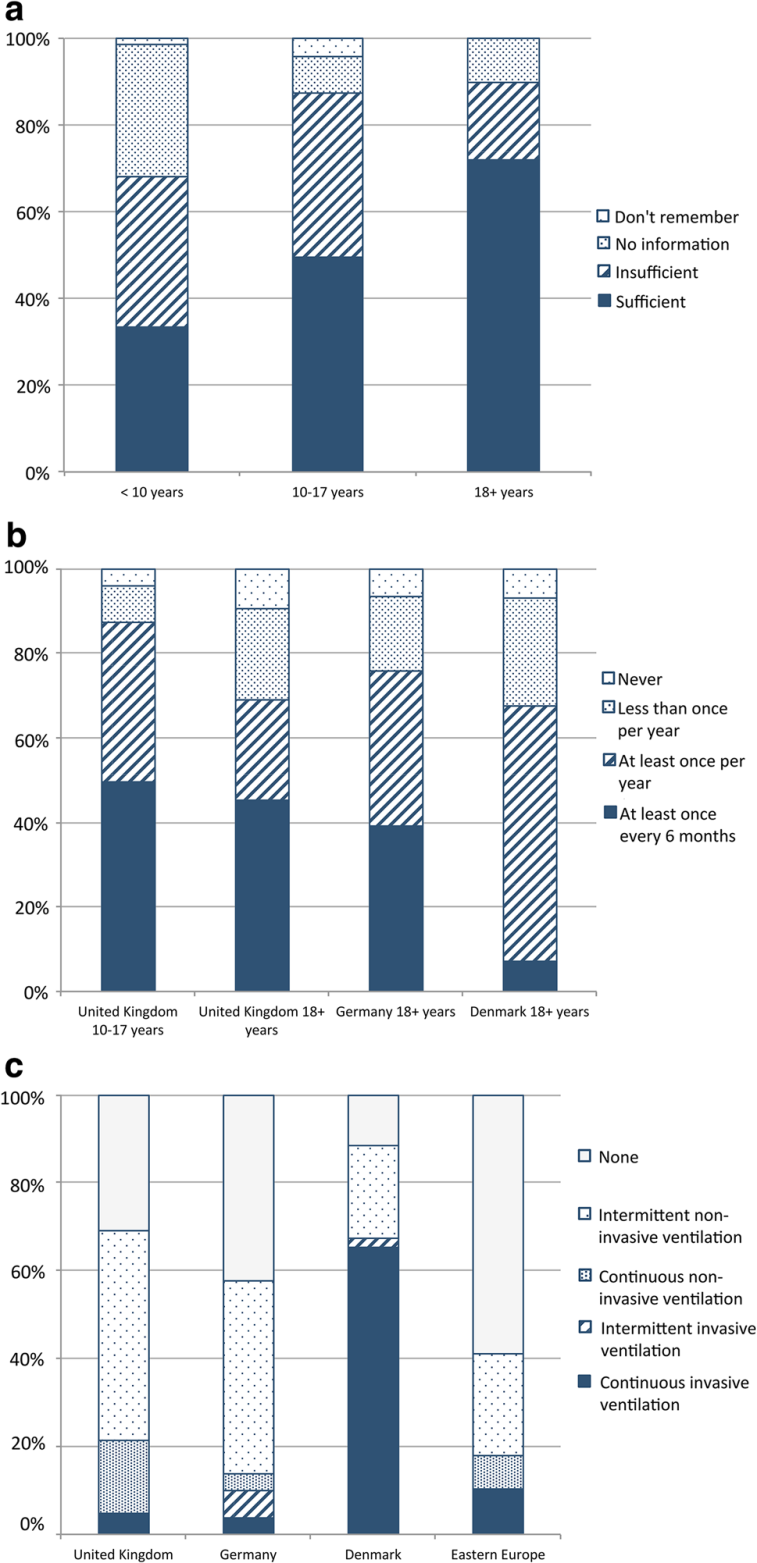
Respiratory care: **a** quality of information provided to different age groups in the UK on respiratory risks, **b** frequency of lung function assessment received by UK adults, UK children, and adults in Germany and Denmark, **c** differences in ventilation practice among adults by country

89.7 % of UK adults received information on respiratory complications, with one-fifth regarding this as insufficient and a further 10.3 % not receiving any information. German adults experienced similar proportions of “sufficient” and “insufficient” information, while Danish adults report higher rates of sufficient information (Table 6). A clear trend was that more adults (89.7 %) received information on respiratory issues than those aged 10–17 (84.0 %) and those under 10 (67.9 %). A more dramatic difference was the proportion who received “sufficient” information: 71.8 % among adults, 51.5 % of those aged 10–17, and 31.3 % among under-10s (Fig 5a).

**Table 6:** Provision of information to adults about respiratory complications by country

Country (valid responses/*n*)	Provision of information about respiratory complications			
	Sufficient	Insufficient	No information	Do not remember
UK (39/42)	28 (71.8 %)	7 (17.9 %)	4 (10.3 %)	0 (0.0 %)
Germany (74/77)	52 (70.3 %)	14 (18.9 %)	5 (6.8 %)	3 (4.1 %)
Denmark (41/43)	35 (85.4 %)	37 (19.5 %)	1 (2.4 %)	1 (2.4 %)
Eastern European countries (36/42)	18 (50 %)	12 (33.3 %)	6 (16.7 %)	0 (0.0 %)

Across all countries a similar proportion of adults had at least annual lung function assessment: 69.0 % (UK), 75.7 % (Germany), and 67.4 % (Denmark). The UK and Germany assessed a greater proportion of these at least every 6 months (45.2 and 39.2 %, respectively) compared to Denmark (7.0 %). With 87.4 % of UK children aged 10–17 reporting at least annual checks, the lower rate of such tests among adults parallels the lower rate of annual cardiac assessment for these two groups (Fig 5b).

Mechanical ventilation was used by 69 % of UK adults, with 47.6 % using intermittent non-invasive ventilation (NIV), 16.7 % using continuous NIV, and 4.8 % continuously ventilated via a tracheostomy. Ventilation practice differed markedly between countries: in the UK, the overwhelming majority (93.1 %) of adults with mechanical ventilation used NIV, while continuous NIV use was also the highest of all countries surveyed. In Germany 88.4 % of ventilated adults used NIV, while in Denmark it was fewer than one in four (23.7 %) with most instead having a tracheostomy. Eastern Europe recorded low rates of ventilation (Fig 5c).

In all countries, invasive ventilation was strongly linked to increased age. The proportion of adults using NIV fell from 89.6 % aged 18–22 to 26.1 % of those aged 33 or older. However, these figures were affected by country, as of the 17 adults aged 33 or older with invasive ventilation, 14 (82.4 %) were in Denmark.

#### Physiotherapy

97.6 % of UK adults received information on preventing scoliosis and contractures, although 14.6 % felt it was insufficient. This is similar to Germany (94.5 and 12.3 %, respectively) while Danish adults all reported being informed and fewer regarded the information they received as insufficient (100 % and 9.3 %). Eastern European adults were less well informed (81.6 and 42.1 %) (Table 7).

**Table 7:** Provision of information about scoliosis and contractures by country

Country (valid responses/*n*)	Provision of information about scoliosis and contractures			
	Sufficient	Insufficient	No information	Do not remember
UK (41/42)	34 (82.9 %)	6 (14.6 %)	1 (2.4 %)	0 (0.0 %)
Germany (73/77)	60 (82.2 %)	9 (12.3 %)	4 (5.5 %)	0 (0.0 %)
Denmark (43/43)	39 (90.7 %)	4 (9.3 %)	0 (0.0 %)	0 (0.0 %)
Eastern European countries (38/42)	16 (42.1 %)	15 (39.5 %)	6 (15.8 %)	1 (2.6 %)

**Fig. 6:**
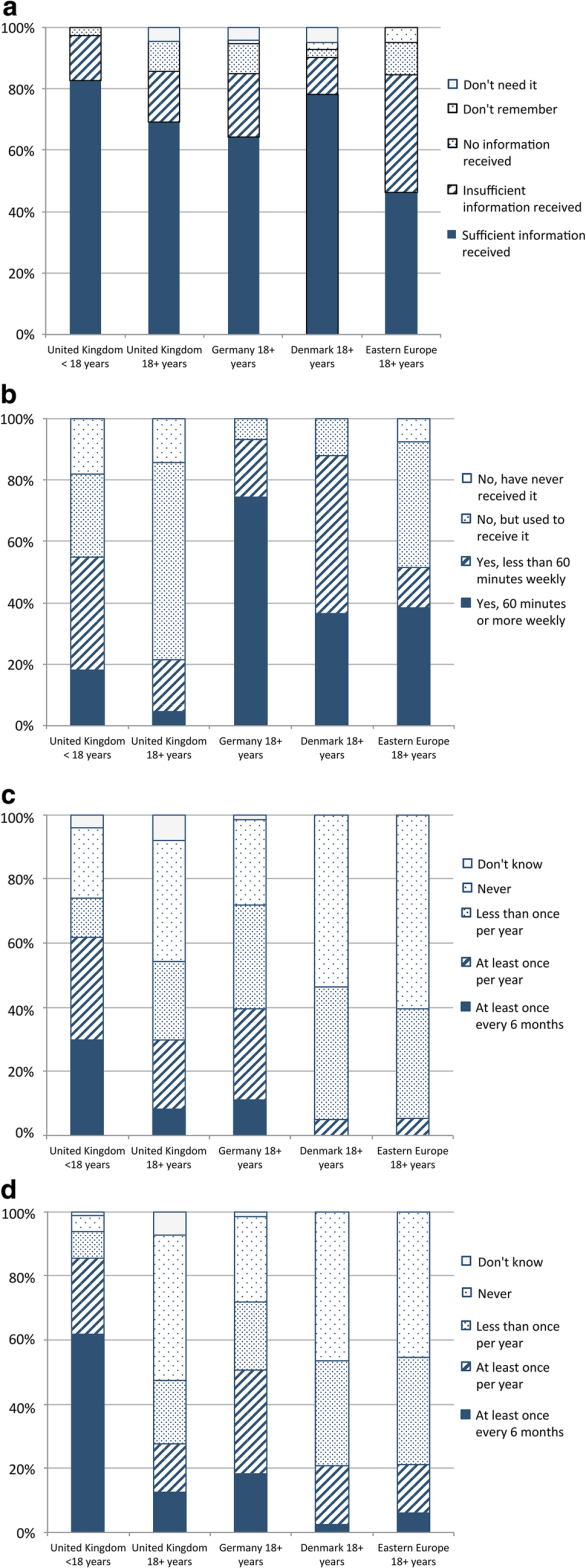
Physical assessment and physiotherapy: **a** instruction received on home stretching, **b** frequency of professional physiotherapy, **c** frequency of spinal assessment, **d** frequency of functional assessment

The proportion of adults who have ever received instruction on home stretching was strikingly similar across countries (84–90 %), but that which believe this to be sufficient varied considerably. Furthermore, notably fewer UK adults regarded information as “sufficient” (69.0 %) than children (82.7 %) (Fig 6a). Access to professional physiotherapy in the UK was very low: few adults (21.4 %) received it at all and only 4.8 % received more than 60 min weekly; the majority (64.3 %) used to receive physiotherapy but no longer receive it (Fig 6b). In contrast professional physiotherapy was received by 93.2 % of German, 87.8 % of Danish, and 51.3 % of Eastern European adults (74.3, 36.6, and 38.5 % over an hour per week, respectively). While UK children had considerably better access to professional physiotherapy than adults (55 %, with 18.1 % at least 60 min weekly), it was still far less than the pediatric cohorts in Germany, Denmark, and Eastern Europe (91.8, 93.3, and 73.5 % overall access, with 55.8, 53.5, and 53.8 % weekly, respectively).

At least annual assessment rates for scoliosis (inspection or spinal radiograph) among adults varied significantly (Fig 6c): 29.7 % (UK), 39.4 % (Germany), and 4.9 % (Denmark), while complete non-assessment was experienced by 37.8, 26.8, and 53.7 % of adults, respectively. Again, scoliosis assessment for UK adults was lower than for UK children. In the UK, 27.5 % of adults received at least annual assessment of functional abilities, and 45 % reported total non-assessment (Fig 6d). Annual adult functional assessment was lower in the UK than Germany (50.7 %), though somewhat higher than Denmark (20.9 %) and Eastern Europe (21.2 %). It was also in stark contrast to UK children, 85.5 % of whom were assessed annually and only 5.2 % not assessed at all.

#### Patterns of multidisciplinary care

These data demonstrated diverse care practices for adults with DMD in the UK. We sought to understand the relationship between different aspects of care, to determine whether certain patients benefited from comprehensive multidisciplinary care. The largest single group of UK adults were the 14 (33 %) receiving at least 6 monthly respiratory and at least annual cardiac assessments. However, we noted a sizeable proportion of UK adults receive less than annual respiratory and cardiac assessments (31 % in each case), and investigated links between these two factors.

**Fig. 7:**
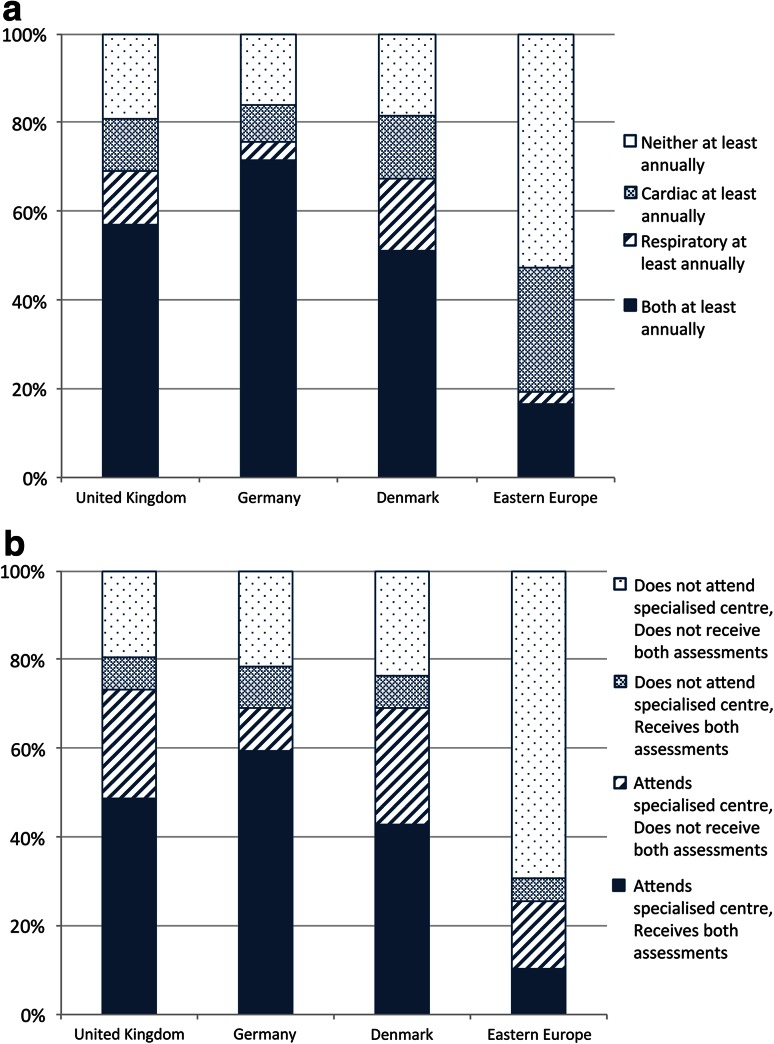
Patterns of multidisciplinary care: **a** relationship between annual cardiac and annual respiratory assessments among adults by country, **b** relationship between attendance at a specialized clinic and annual assessments among adults by country

There was considerable overlap between those who received both cardiac and respiratory assessments at least annually (24 individuals, 57.1 %), while eight individuals (19.0 %) received neither (Pearson’s Chi square, *p* < 0.005). Similar correlations were observed in Germany (71.6 % both and 16.2 % neither, *p* < 0.001) and Denmark (51.2 % both and 18.6 % neither, *p* < 0.05). Across all Western European countries, these data were significant to *p* < 0.001 (Fig 7a).

Investigating the relationship between specialized clinic attendance and annual assessments of both respiratory and cardiac function revealed that the largest group was those who were seen at a specialized clinic (20 individuals, 48.8 %). Conversely, only three adults who did not attend a specialized clinic received such frequent assessments. However, of 18 UK adults not seen at least annually for both assessments, a slight majority (10, 55.6 %) did attend a specialized clinic (*p* < 0.05).

A similar pattern emerged in Germany and Denmark, and is significant in both. Across Western Europe, this pattern is significant to *p* < 0.001. In Eastern Europe, a similar pattern is observed (*p* < 0.05). The single largest group in Western Europe were 82 individuals (52.2 %) both seen at a specialized clinic and receiving annual cardiac and respiratory assessments, while in Eastern Europe it is 27 adults (69.2 %) who are neither seen at a specialized clinic nor receive both annual assessments (Fig 7b).

## Discussion

To our knowledge, we have conducted the largest survey of DMD patients to date, and this study highlights considerable differences between the care received by adults and children in the UK. It also reveals distinctive care practices and differing levels of societal integration of adults with DMD between the UK and other European countries.

We are mindful of limitations including incomplete coverage of the DMD population and the study design. While the CARE-NMD was unprecedented in size and had a good response rate (53.7–77.1 % in Western Europe), the scarcity and life-limiting nature of DMD resulted in relatively few adult respondents overall. Once stratified by country and other attributes, this precluded advanced statistical analyses, especially in Eastern Europe. Furthermore, questionnaire distribution via opt-in patient registries will likely have resulted in selection bias: registries tend to attract patients who are most engaged with the DMD community, and who are aware of and more inclined to campaign for best practice care. However, with shortcomings in treatment apparent even for this better-informed population, we feel this does not detract from our findings.

The study design also precluded the analysis of care of those who have died (unlike a retrospective case review or audit) which might identify patterns of care leading to specific clinical outcomes. We did not ask respondents to name the clinic(s) they attended, as we felt this would have reduced participation due to the possibility of individual identification. However, this also prevents us from identifying specific examples of good or poor practice. Finally, self-report questionnaires may not accurately reflect clinic provision, and cannot account for non-attendance of patients registered with a center that otherwise provides comprehensive care. In spite of these limitations, we believe that this study provides a valuable insight into the current care received by adults with DMD in the UK.

Care for adults in the UK is considerably less comprehensive than for children, and fewer adults attend specialized neuromuscular clinics. Adults are also less likely to be satisfied with their care and considerably less likely to have their functional abilities assessed or to receive professional physiotherapy than children. A large proportion report that they formerly had access to professional physiotherapy, supporting previous findings that such access is especially poor among adults with chronic conditions in the UK [13, 17, 18], and that there is a particular problem at transition from childhood to adulthood. This conclusion is reinforced by other results from this survey. Most notably adults are less likely than those aged 10–17 to receive at least annual echocardiograms and lung function checks as recommended in the care guidelines to all patients aged over 10 years. In spite of an increased need, best practice care provision thus actually declines as these men reach adulthood. This lends weight to previous studies which found that transition, and especially transfer to adult services, is a critical point when access to comprehensive care is lost in DMD [12, 19, 20] and other chronic conditions [21].

Compared to elsewhere in Europe, care for adults with DMD in the UK is inconsistent. Although annual and 6 monthly attendance of adults at specialized clinics in the UK is higher than elsewhere, the proportion of adults who do not attend one at all is also the highest of the Western European countries surveyed (22 %). The UK also has a very low rate of social inclusion, with the highest proportion of adults living with their family in Western Europe and the lowest proportion living independently and participating in employment or education. These data support previous findings, both on the UK experience [16, 19] and how it compares to independent living in Denmark [11]. The UK has by far the lowest access to adult physiotherapy of any country surveyed, most likely because long-term adult physiotherapy is not commonly funded through the National Health Service. It also reflects a very low rate of access to professional physiotherapy in the UK generally, with the pediatric cohort also the lowest of all countries. For adults, maintaining upper extremity function helps to preserve functional abilities which enable independence (such as wheelchair and assistive device use), which alongside preventing painful contractures and stiffness can improve quality of life [8, 22].

In other areas, UK care practices for adults with DMD are more positive. The rate of instruction in home stretching is comparable to Germany and Denmark, which may offset the lack of professional physiotherapy. Although a sizable minority of adults does not receive the recommended frequency of cardiac and respiratory checks, these results are broadly similar to elsewhere in Western Europe and are considerably better than in Eastern Europe. The UK also has the highest rate of prophylactic administration of cardiac medication among adults (42.9 %) and children aged 10–17 (14.9 %) of all countries surveyed, approximately double that in Germany and over three times that in Denmark.

UK adults also spent fewer nights in hospital overall and per patient, for both planned and unplanned admissions, than adults elsewhere in Western Europe. The single largest cause of admissions was unplanned respiratory crises, which accounted for more than three-quarters of unplanned admissions in the UK and Germany. In spite of their advanced age, the proportion of Danish unplanned nights in hospital due to respiratory crises is very low and considerably lower than in the UK. Reducing unplanned hospital admissions has been the subject of significant research, as they are problematic for the individual and costly to the health system [23, 24]. Previous research has identified problems associated with unplanned admissions in neuromuscular conditions [25], and a recent audit suggested that up to a third may be preventable [26, 27]. These results suggest that although the UK records a low absolute number of nights spent in hospital for adults with DMD, more could be done to reduce unplanned respiratory admissions, particularly as the study design may also underestimate the number of nights attributable to this cause as it does not record those experienced by patients who died following such admissions.

It is striking that almost all ventilated UK adults use non-invasive NIV, while in Denmark most have a tracheostomy, likely due to long-standing differences in cultural attitudes and medical practices toward invasive ventilation. The UK has the highest rate of continuous NIV of any country, while Denmark appears to switch from intermittent NIV to invasive ventilation. However, these data also show that invasive ventilation is strongly linked to the age of the respondent, and as the Danish cohort includes many more older men (48.8 % are over 30 vs. 9.5 % in the UK, with more than 80 % of those aged over 33 being Danish), it is not possible to say with certainty whether these differences are solely due to cultural or healthcare system preference. It is also notable that no clear pattern emerged between UK unplanned admissions and respiratory care practices, nor did age appear to be the determining factor in unplanned adult admissions in the UK or elsewhere in Western Europe.

We demonstrate a statistically significant link between frequency of cardiac and respiratory assessments, and between regular attendance at a specialized center and more frequent assessment. Given significant mortality associated with cardiac and respiratory failure in this population, and the importance of heightened vigilance for these complications, best practice care for adults with DMD may be best delivered in a specialized neuromuscular clinic. This supports findings from cystic fibrosis (CF) and other life-limiting chronic conditions [28, 29]. With most adults who provide a reason for non-attendance at a specialized clinic citing the distance they would need to travel, we suggest that the difficulties associated with traveling as a wheelchair and ventilator-dependent adult may lie behind this non-attendance.

We also found that adult attendees of specialized clinics are more likely to be satisfied with their care than non-attendees, a pattern also observed across Europe. Attendees of specialized clinics are also better informed of the disease and its complications. This is unsurprising, as a core rationale for such centers is to concentrate and develop specialist expertise and provide tailored information to patients [30]. Medical professionals outside these centers may not see sufficient patients to be fully aware of best practice care, or may be less inclined to provide this information to patients than staff at a specialized clinic. Those aged 10–17 are more likely to receive sufficient information about cardiac and respiratory risks than the under-10s, probably due to the increased need for respiratory and cardiac intervention from the second decade. The low proportion of adults receiving information on steroid treatment likely reflects its relatively recent introduction as part of best practice care [3], a conclusion supported by a disparity in the mean age of those attending a specialist center who did not receive information on steroids (27.2 years) and those who did (21.6 years).

The data we present expands upon and quantifies previous studies which have noted the paucity of best practice adult care provision for DMD and the difficulties experienced at transition. It emphasizes the need for a comprehensive, co-ordinated programme of multidisciplinary care and social support for the growing number of adults living with DMD in the UK. Until recently, there has been no systematic commissioning for adult neuromuscular care in the UK, though this is now part of the specialized commissioning remit [31]. With nearly a third of UK adults not receiving the recommended cardiac and respiratory checks, there is significant room to improve care for this population, and some areas require considerable attention to ensure that adults with DMD in the UK receive care in line with current best practice recommendations.

## Footnotes

1 With the exception of the 2–4 year age group, where only the parent report was distributed.

2 Bulgaria, the Czech Republic, Hungary, and Poland.
